# The fitness burden imposed by synthesising quorum sensing signals

**DOI:** 10.1038/srep33101

**Published:** 2016-09-12

**Authors:** A. Ruparell, J. F. Dubern, C. A. Ortori, F. Harrison, N. M. Halliday, A. Emtage, M. M. Ashawesh, C. A. Laughton, S. P. Diggle, P. Williams, D. A. Barrett, K. R. Hardie

**Affiliations:** 1 School of Life Sciences, Centre for Biomolecular Sciences, University of Nottingham, University Park, Nottingham, NG7 2RD, United Kingdom; 2 Centre for Analytical Bioscience, School of Pharmacy, University of Nottingham, Nottingham NG7 2RD, United Kingdom

## Abstract

It is now well established that bacterial populations utilize cell-to-cell signaling (quorum-sensing, QS) to control the production of public goods and other co-operative behaviours. Evolutionary theory predicts that both the cost of signal production and the response to signals should incur fitness costs for producing cells. Although costs imposed by the downstream consequences of QS have been shown, the cost of QS signal molecule (QSSM) production and its impact on fitness has not been examined. We measured the fitness cost to cells of synthesising QSSMs by quantifying metabolite levels in the presence of QSSM synthases. We found that: (i) bacteria making certain QSSMs have a growth defect that exerts an evolutionary cost, (ii) production of QSSMs negatively correlates with intracellular concentrations of QSSM precursors, (iii) the production of heterologous QSSMs negatively impacts the production of a native QSSM that shares common substrates, and (iv) supplementation with exogenously added metabolites partially rescued growth defects imposed by QSSM synthesis. These data identify the sources of the fitness costs incurred by QSSM producer cells, and indicate that there may be metabolic trade-offs associated with QS signaling that could exert selection on how signaling evolves.

Communication systems are widespread in plants, animals and microorganisms. For true communication (signaling) to evolve, signals must transfer information that benefits both the signaler and the receiver. Whether signals are visible, acoustic or chemical in nature, their production implies a cost to the emitter^1^, but these costs are often difficult to measure experimentally^1^,^2^,^3^,^4^. Many bacterial species communicate using small diffusible signals to co-ordinate social behaviours in a process termed quorum sensing (QS)^5^,^6^. QS signaling molecules (QSSMs) are synthesized inside the bacterial cell and released into the surrounding environment. Once accumulated to a threshold concentration, the QSSMs drive the expression of genes encoding public goods and other social behaviors that benefit the surrounding population of cells. Previous work has shown that there are fitness costs (e.g. metabolic ‘tradeoffs’^7^) associated with producing QS-regulated public goods, and that these costs are significant enough for non-producing cheats (i.e. mutants that can respond to QSSMs, but do not make them) to evolve and spread in populations^8^,^9^,^10^. Evolutionary theory has also predicted that the production of the QSSMs themselves should also incur a fitness cost. For example, the ‘handicap principle’ suggests that if a signal is reliable, then it must carry some cost to the signaler^1^,^11^,^12^,^13^. Previously such a cost has been implied by growth deficiencies in producing strains^10^. This study sought to identify the source of this cost by examining the fitness consequences of producing QS signals in detail. Any fitness costs are presumed to be a drain on metabolites. Keller and Surette estimated that production of each of three well-studied QSSM classes (oligopeptides, *N*-acylhomoserine lactones (AHLs) and Autoinducer-2 (AI-2) impose a metabolic cost of 184 ATPs, 8 ATPs and 0-1 ATP respectively^2^. We therefore set out to determine experimentally whether there are metabolic consequences for QSSM synthesis.

We chose to work with the two dedicated QSSM LuxI-type synthases (LasI and RhlI) that produce AHLs in the opportunistic, multi-antibiotic-resistant pathogen *Pseudomonas aeruginosa*. LasI synthesizes long chain AHLs (predominantly *N-*(3-oxododecanoyl)-L-homoserine lactone (OC_12_-HSL)). RhlI primarily synthesizes the short chain AHL (*N-*butanoyl-L-homoserine lactone (C_4_-HSL)). *P. aeruginosa* incorporates these AHL synthases into a complex hierarchical QS network which controls virulence factor production and thus pathogenicity in plants and animals (including humans)^14^. LasI and RhlI were each introduced into a heterologous host, *Escherichia coli,* that does not naturally produce AHLs. We reasoned that it is important to determine whether there are fitness costs specifically associated with QSSM production *per se*. A future challenge remains with respect to understanding the fitness burden of a complete QS system in its natural, adapted host. In this context, it would be possible to consider the added cost of responding to the QSSM: this may in turn be integrated into a complex regulatory network or have pleiotropic effects unlinked to the response to signals.

Synthesis of AHLs depends on the availability of precursors i.e. an appropriately charged acyl carrier protein (acyl-ACP) and *S*-adenosyl-L-methionine (SAM)^15^,^16^,^17^,^18^. The donation of the acyl group from acyl-ACP to the amine of SAM results in the formation of the AHL and release of 5′-methylthioadenosine (MTA)^15^,^16^,^18^. Both LasI and RhlI use SAM, but link it to a different fatty acid. We can assume they are approximately catalytically equivalent, although kinetic data is only available for RhlI^17^,^18^. As the major methyl donor in eubacterial, archaebacterial and eukaryotic cells, SAM is a critically important metabolite^19^,^20^. The availability of SAM and relative flux through the activated methyl cycle (AMC: to which SAM contributes, see Supplementary Fig. S1) significantly impacts upon central metabolism and influences cell fitness. This has been well documented in the context of two AMC enzymes, LuxS and Pfs^21^,^22^,^23^,^24^,^25^,^26^.

Here we test the metabolic costs (by measuring AMC-linked metabolite levels) and fitness costs (by monitoring growth) of making an AHL QSSM in the heterologous host, *E. coli.* We show that (i) signal production can cause a growth defect that imposes a fitness disadvantage in mixed populations; (ii) signal production is negatively correlated with intracellular substrate concentrations; (iii) heterologous signal production negatively impacts native signal production; and (iv) supplementation with exogenously added metabolites partially rescues growth defects imposed by signal synthesis. Our findings demonstrate that the fitness cost of generating the QS signals required for co-ordinated social behaviour in bacteria can be substantial.

## Results

### Production of chemical signals used for social communication compromises cell fitness

**Figure 1:**
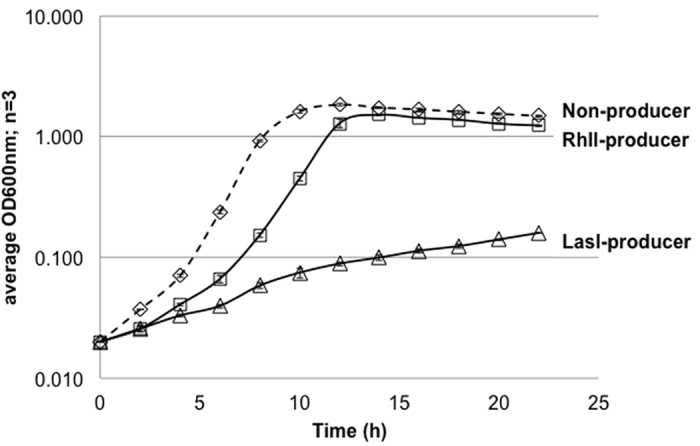
QSSM synthesis alters the fitness of bacteria. Three *E. coli* strains, the AHL non-producer MG1655(pME6032), LasI-producer MG1655(pME-*lasI*), and RhlI-producer MG1655(pME-*rhlI*) were each inoculated into 125 ml MMM + tetracycline + IPTG and grown with shaking at 37 °C in 500 ml. OD_600_ measurements are given as means ± standard deviations for three independent cultures.

Bacterial populations can communicate using QSSMs, and it has been shown that QS responses impose a fitness cost^27^. To test whether QSSM synthesis is also metabolically costly, the genes encoding QSSM synthases LasI and RhlI were expressed from the low copy number shuttle vector pME6032 in *E. coli*. Having hypothesized that QSSM synthesis would compromise cell fitness, the growth profiles of the strains were compared in both minimal (MMM; Fig. 1) and rich (LB; Supplementary Figs S4 and S5) media. Bacteria containing the empty plasmid pME6032 (referred to as non-producer) had no detrimental effect on growth. In LB medium containing abundant nutrients, there was a slightly slower initial growth rate and lower final density upon induction of the AHL synthase LasI, demonstrating a fitness cost and by extension a potential metabolic cost (Supplementary Fig. S4). More drastic effects on growth were observed in MMM. This is in line with observations that disruption of one of the metabolic cycles feeding into AHL synthesis (the AMC) is more readily reflected in growth defects in defined media limited for the sulphur sources that feed into this pathway^21^,^24^,^26^,^28^,^29^. The RhlI-producer MG1655(pME-*rhlI*) did not grow as well as the empty vector control initially (Fig. 1), but achieved the same final population density. In accordance with the higher level of LasI production (indicated by readily detectable levels of LasI using Coomassie staining of SDS PAGE while RhlI could only be detected by the more sensitive immunoblotting technique: Supplementary Fig. S2), it had an even more dramatic effect on growth, with the LasI-producer MG1655(pME-*lasI*) growing much more slowly than the non-producer, and failing to achieve a population size (OD_600_) equivalent to the other strains within 22 h in MMM (Fig. 1).

The production of the major cognate QSSMs in *E. coli* culture supernatants by LasI and RhlI (OC_12_-HSL and C_4_-HSL respectively) was confirmed by thin-layer chromatography (TLC) (data not shown) with quantification using sensitive LC-MS/MS (Supplementary Fig. 2). In line with an absence of inhibition of *E. coli* growth inflicted by exogenous addition of these concentrations of QSSMs (Supplementary Fig. S6; up to 800 μM), no defect in growth was observed in rich medium with the RhlI-producing strain (Supplementary Fig. S5), and the growth of the LasI-producer was only marginally reduced in rich media (Supplementary Fig. S4). The masking of the overall cost of producing signals by growth in rich medium suggests that it may, at least in part, derive from an energetic cost that could be provided by exogenous metabolites present in LB. To determine the lower limit for the level of QSSM production that results in a cell fitness cost, IPTG concentrations were titrated down to reduce the amount of RhlI or LasI produced (Supplementary Fig. S7). At the point where QSSMs were barely detectable (0.004 mM for OC_12_-HSL and 0.0008 mM for C_4_-HSL), all strains grew at a similar rate and reached a comparable population density at stationary phase.

### Mutations that prevent signal production rescue bacterial growth defects

It is possible that the observed growth defects may be due to the burden of protein overproduction in producers, rather than signal synthesis. To investigate this possibility, a number of mutations were introduced into RhlI and LasI that resulted in altered or abolished AHL production (Supplementary Figs S4 and S5). The LasI/RhlI amino acid residues mutated were chosen as described below.

The structures of the AHL synthases LasI and EsaI were used to model the predicted structure of RhlI (Supplementary Fig. S3) in order to identify key residues predicted to be involved in catalysis. These were used as targets for parallel site directed mutagenesis of LasI and RhlI. The catalytic residues chosen for mutagenesis were based on those identified in a previous study^30^ that screened the activity of a collection RhlI mutations. LasI residues F27 and W33 are important for SAM binding, and S103 appears to participate within a cluster of other residues to maintain tertiary structure interactions and may also perform a catalytic function^31^. Changes were also made to residues that may alter a specific property of the active site (see Supplementary Table S2), and to R23 of LasI since we predicted it is involved in catalysis. All the residues selected mapped to the vicinity of a pocket hypothesized to be the active site (Supplementary Fig. S3).

The AHL synthase mutants that could be overproduced as a protein of the predicted size were selected for further study (Supplementary Figs S4, S5). Wild type levels of QSSMs were produced by the LasI mutants F27Y and S103A (Supplementary Fig. S4). Similarly, mutation of S103 to either A or V in RhlI did not significantly lower total AHL production (Supplementary Fig. S5). With the exception of LasI F27L, the other AHL synthase mutants lost the ability to synthesize AHLs. All the mutants able to synthesize AHLs, except LasI S103A, did so in relative proportions that resembled the profile of the wild type AHL synthase. Significant concentrations of C_4_-HSL, which were not observed with wild type LasI, accounted for 20% of the AHLs made by LasI S103A (Supplementary Figs S4 and S5).

Importantly, only AHL synthase mutants that retained the ability to synthesize AHLs showed growth inhibition, indicating that the enzymatic activity of the QSSM synthases resulted in a fitness burden (Supplementary Figs S4 and S5).

### Increased levels of signal correlated with reduced intracellular concentrations of substrates used to make them

**Figure 2:**
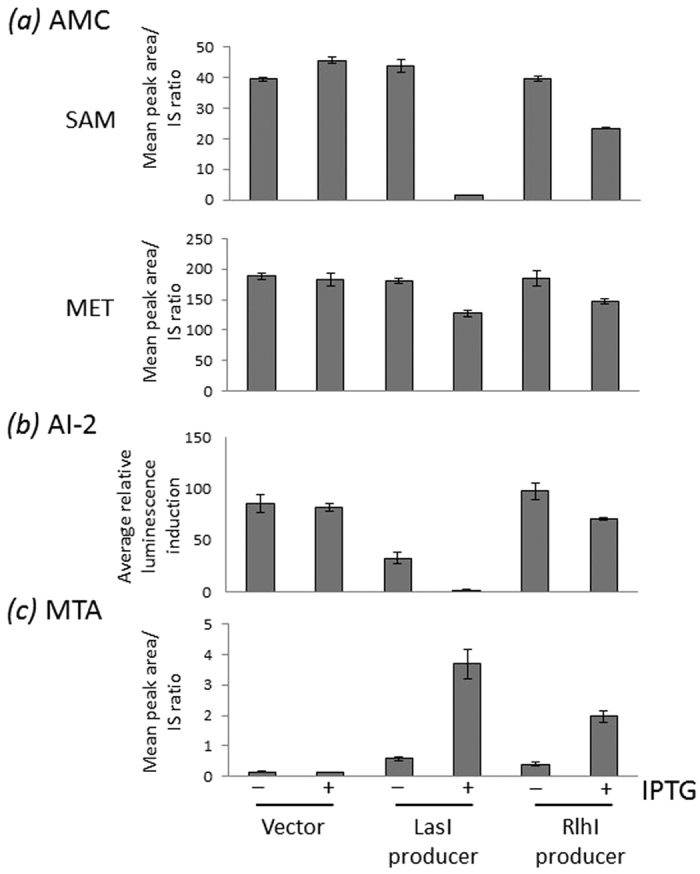
AHL synthases reduce levels of AMC-linked metabolites including the native QSSM AI-2, whilst intracellular MTA levels increase. Panel (a) Metabolite levels were determined in three *E. coli* strains MG1655(pME6032), MG1655(pME-*lasI*) and MG1655(pME-*rhlI*) grown in LB + tetracyline with and without IPTG. Intracellular accumulation of SAM, SAH, SRH, HCY and MET were determined by LC-MS analysis. SAM and MET levels represent the most and least profound modulation respectively. Peak area corresponding to each compound was divided by the peak area of the appropriate internal standard (IS) for normalisation. Panel (b) Intracellular accumulation of MTA in similarly grown MG1655∆*pfs*(pME6032), MG1655∆*pfs* (pME-*lasI*) and MG1655∆*pfs* (pME-*rhl*). Panel (c) Spent culture supernatants were prepared from MG1655(pME6032), MG1655(pME-*lasI*) and MG1655(pME-*rhlI*) grown in LB + tetracylcine with and without IPTG until an OD_600_ of 0.75, 0.80 and 0.88. The average bioluminescence induced by AI-2 reporter *V. harveyi* strain after 2 h incubation is shown. Metabolite levels for a single experiment are shown, although the experiment has been repeated three times with similar results.

As producers synthesize AHLs from specific metabolic substrates, we hypothesized that the growth defect observed may arise as a consequence of introducing metabolite-consuming enzymes. One of the metabolites central to the AMC pathway, SAM, is a substrate for AHL synthesis. We therefore determined using LC MS/MS the profiles of the AMC metabolites (SAM, SAH, SRH, HCY and MET) to test our hypothesis. In late exponential phase cells, the level of each AMC metabolite measured was reduced following signal production (Fig. 2a). Metabolite concentrations were more dramatically reduced in LasI-producers than in RhlI-producers. The metabolite that exhibited the greatest percentage concentration change (97% in LasI-producers, 45% in RhlI-producers) was SAM (Fig. 2a, Supplementary Table S4). Enzyme activity was key to these metabolic perturbations because no fall in metabolite levels was observed in an AHL synthase mutant lacking the ability to synthesize AHLs (Supplementary Figs S4 and S5).

### The production of foreign signals negatively impacts the production of a native signal

One of the reactions of the *E. coli* AMC is catalysed by LuxS, and leads to the generation of AI-2. AI-2 acts as a QSSM, e.g. that stimulates production of bioluminescence by *Vibrio harveyi*. In *E. coli*, inactivation of *luxS* has a pleiotropic effect. It is not clear what signalling role LuxS plays due to variations in strains and mutagenic strategies, although it can influence the virulence of pathogenic *E. coli*^32^,^33^. To determine the influence of a foreign QSSM synthase upon the production of a native QSSM, the amount of AI-2 produced in culture supernatants was measured. The synthase genes caused a reduction in the levels of AI-2 detected, with AI-2 levels falling by 12% in C4-HSL-producers and by a massive 54-fold in 3OC_12_-HSL-producers (Fig. 2b).

### The fitness cost of QSSM synthesis is partly due to the production of toxic side products

AHL-synthase catalyzed production of AHLs generates a second product, MTA, which could potentially have metabolic consequences since it is a potent feedback inhibitor of polyamine biosynthesis^34^. To determine if MTA accumulates as a result of AHL synthesis, intracellular accumulation of MTA was monitored (Fig. 2c). These measurements were conducted in a defined *E. coli* Δ*pfs* mutant in parallel with MG1655 because, in addition to catalysing the detoxification of SAH to SRH in the reaction preceding LuxS in the AMC, Pfs can act as an MTA nucleosidase^35^, and thus potentially degrade MTA faster than we can measure it. Although MTA may not accumulate to detectable levels in MG1655, if the Δ*pfs* mutant were to show higher levels there would be the potential for transient MTA accumulation that could impact upon cell fitness. As predicted, MTA levels in *E. coli* MG1655 were highly variable (Supplementary Fig. S8), while in the absence of Pfs, accurate and reproducible levels of MTA were determined (Fig. 2c). Furthermore, higher basal levels of MTA were detected in the *E. coli* Δ*pfs* mutant compared with *E. coli* MG1655. In both genetic backgrounds, there was a clear trend indicating that in the presence of an AHL synthase, MTA accumulated. Induction of *lasI* in the *E. coli* Δ*pfs* mutant resulted in a 26-fold increase in MTA whilst induction of *rhlI* caused a 14-fold increase in MTA compared to the equivalent empty vector control.

### Supplementation with exogenously added metabolites partially rescued growth defects imposed by QSSM synthases

**Figure 3:**
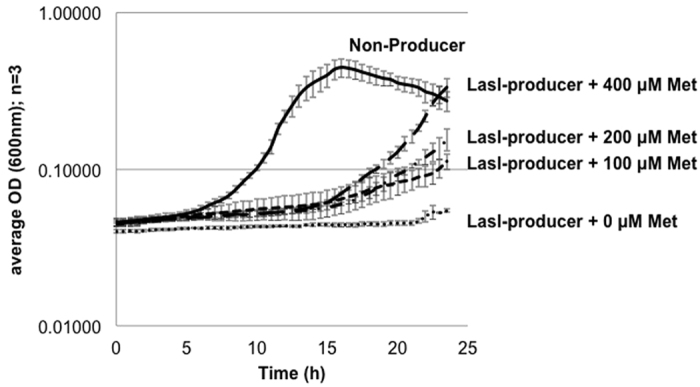
Exogenous methionine addition partially rescues the growth defect imposed by QSSM synthases. MG1655(pME6032) (solid line) and MG1655(pME-*lasI*) (broken lines) were inoculated into MMM + tetracycline, IPTG induced and methionine was added to cultures of MG1655(pME-*lasI*) at time zero. Selected methionine concentrations (μM) are shown. Strains were grown in an automated microplate reader (TECAN Infinite F200) and changes in cell density (OD_600_) monitored. The data are means ± standard deviations for three independent experiments.

As LasI and RhlI production impedes the growth of *E. coli* and drains away AMC metabolites, the possibility that the exogenous addition of a metabolite that feeds into the AMC could restore growth was investigated. Despite an approximate 14-h delay, exogenous methionine promoted the growth of LasI-producers in a concentration dependent manner (Fig. 3), indicating that the fitness cost of QSSM synthases can be partially rescued by replenishing the substrates for these enzymes, thus the cost is likely to be at least partly energetic. Automated sampling facilitated the measurement of growth throughout the entire growth curve. This necessitated growth in small volumes in a microtitre plate which generated overall kinetics that differed from cultures grown in shaking flasks at larger volumes such as depicted in Supplementary Fig. S4. Parallel exogenous methionine supplementation in flasks also partially rescued growth defects (data not shown).

### A QSSM imposed growth defect confers a fitness cost in mixed populations

**Figure 4:**
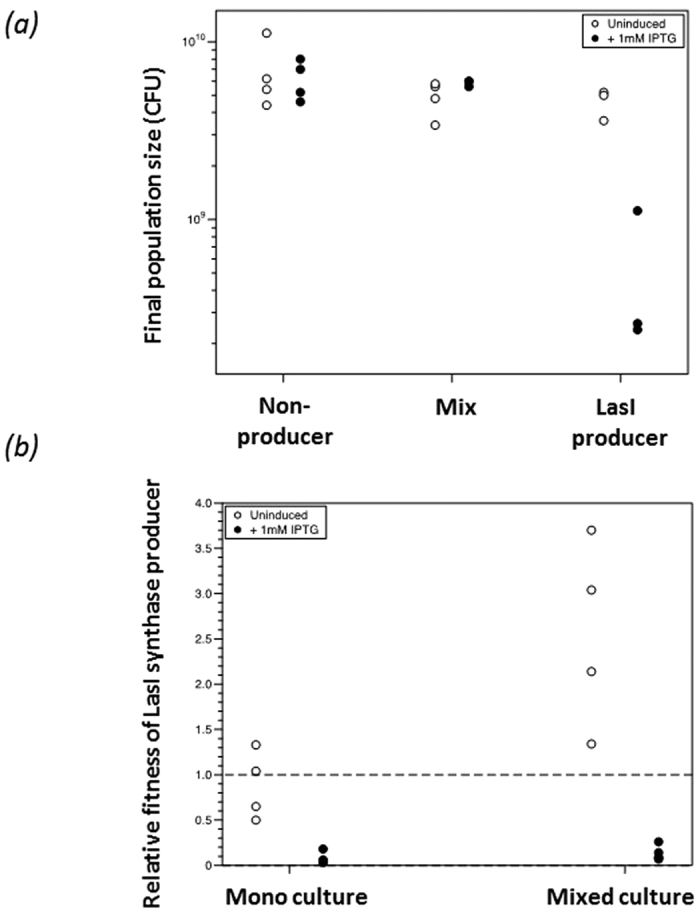
LasI imposes a fitness cost within a mixed population. *E. coli* MG1655(pME6032), *E. coli* MG1655(pME-*lasI*) or a 1:1 mixture of the two strains were grown. Colony counts were used to calculate the total population density. To determine the population density of each strain in mixed populations, cfu was assessed (Panel a). The evolutionary fitness of *E. coli* MG1655(pME-*lasI*) relative to *E. coli* MG1655(pME6032) in mixed populations is plotted (Panel b). When the two genotypes have equal fitness, *v* = 1. Values of *v* < 1 reflect being outcompeted by MG1655(pME6032 and values >1 indicate that MG1655(pME-*lasI*) outcompetes MG1655(pME6032). The relative fitness of *E. coli* MG1655(pME-*lasI*) in pure culture was calculated by randomly pairing pure MG1655(pME-*lasI*) and MG1655(pME6032) populations within IPTG treatment.

Having demonstrated the fitness cost of producing communication signals, we tested whether this was likely to generate a selective advantage in the absence of a beneficial public goods production response, that could impose evolutionary pressure in conditions more closely mimicking the natural environment where different bacterial strains co-exist in mixed populations. This is particularly important since QSSMs are themselves diffusible public goods available to non-producers. To do this, a QSSM producer and non-producer were grown singly and together, and their relative fitness assessed by ANOVA (Fig. 4).

The total population density was affected by genotype (non-producer, LasI-producer or mix; ANOVA: F_2,18_ = 29.6, *p* < 0.001) and presence/absence of IPTG to induce the AHL synthase (ANOVA: F_1,18_ = 13.4, *p* = 0.002). Moreover, the effect of IPTG depended on population (interaction F_2,18_ = 18.2, *p* < 0.001). As shown in Fig. 4a, in the absence of IPTG, there were no significant differences in the total densities reached by the non-producer, LasI-producer or mixed populations (Tukey HSD tests, *p* > 0.47), but in the presence of IPTG, LasI-producer growth was around one log lower than either the non-producer or the mix (*p* < 0.001). Thus, adding IPTG decreased growth of LasI-producer, but did not affect the other two populations. Dropping the single outlier from the data set did not affect these results.

The relative fitness of LasI-producer depended on whether the two strains were grown in pure culture or in a mixture (ANOVA: F_1,12_ = 12.9, *p* = 0.004), on the presence of IPTG (ANOVA: F_1,12_ = 78.6, *p* < 0.001) and on the interaction between culture condition and IPTG (ANOVA: F_1,12_ = 6.96, *p* = 0.022). In the absence of IPTG, the two strains grew equally well in pure culture (Fig. 4a) and post-hoc *t*-tests showed relative fitness not significantly different from 1: *p* = 0.062) although the LasI-producer had a fitness advantage in mixed culture (Fig. 4b) *p* < 0.001). In the presence of IPTG, the relative fitness of LasI-producer was <1 regardless of culture condition (*p* < 0.001).

## Discussion

A number of studies have used bacterial QS linked phenotypes to test social evolution theory because QS controls the production of costly ‘public goods’ and this creates a drain on the fitness of the cells^6^,^7^,^9^,^10^,^36^,^37^,^38^,^39^,^40^. Although theory suggests that signal production can be costly^7^,^11^, and growth deficiencies have implied a cost^10^, the source of this cost has not previously been established. Here we provide direct evidence identifying the source of the costly metabolic burden imposed on cells by the production of QSSMs. Specifically we found that there was a two-fold cost of signal production under resource-limited conditions due to (a) a metabolic trade-off^7^ which slowed down bacterial growth when signals were produced, and (b) the accumulation of toxic side products that further limited bacterial growth.

### Mechanistic insights

Our findings provide experimental support for the theory of Keller and Surette^2^, who calculated the metabolic cost of QSSM synthesis in terms of the ‘energy currency’, ATP. Quantification of AHL production in our experiments, as expected, revealed that the most abundant AHLs were the cognate QSSMs, with pME-*lasI* and pME-*rhlI* directing the production of OC_12_-HSL (~30 μH, 71%) and C_4_-HSL (~40 μH, 95%) respectively at the highest IPTG concentration used (Supplementary Fig. S2). These quantities are broadly in line with AHL production in *P. aeruginosa,* where concentrations of 0.5–15 μM for OC_12_-HSL and 5–31 μM for C4-HSL have previously been reported^41^,^42^,^43^,^44^.

The approximate energetic costs of these AHL levels was crudely calculated. It has been estimated that an *E. coli* cell contains 12.1 billion ATP molecules^45^. Using a value of 30 μM corresponding to the highest concentration of OC_12_-HSL observed at 1 mM IPTG, we estimate the total cost of ATP production to be significant at ~7% of the total ATP. This is in line with metabolic perturbations caused by QS^46^, but should in future be experimentally investigated to accurately reflect the metabolite turnover in the conditions studied.

The QSSM levels produced in our study only created a marginal growth defect in rich medium (Supplementary Figs S4 and S5). This supported our assumption that they would not be toxic, which was based on the use of *E. coli* AHL bioreporters. Reduction of signal synthesis by titration of IPTG, reduced AHLs to undetectable levels and concomitantly repaired the observed growth defects (Supplementary Fig. S7). The observation that growth defects resulting from the introduction of AHL production were more prominent in MMM supports our hypothesis that the metabolic drain of AHL production would occur via the AMC since previous studies have shown that disruption of the AMC is more readily reflected in growth defects in defined medium limited for the sulphur sources that feed into this pathway^21^,^24^,^26^,^28^,^29^.

A critical question arises regarding the biological relevance of the experimental set up of the study, since it uses medium copy (approximately 15) plasmids with an inducible p*trc* promotor rather than a single chromosomal copy of the QSSM synthase encoding genes under the control of their native promoters. The experiments were conducted in this manner to provide us with control over signal production, enabling full induction of the signal at a specified point with the primary aim of determining if signal production can incur a fitness cost. It is possible that even a cost equivalent to a small percentage of what we measured could have a significant impact in natural populations and provide a selective pressure for bacterial evolution. The genes studied here are not commonly plasmid-borne, but QS has been extensively studied in this context^15^,^27^,^30^,^35^,^38^,^47^, as has the activity and impact of many microbial genes including those that would create a fitness cost that might induce compensatory changes in the native genetic background. Furthermore, the AHLs studied here have been added exogenously to *E. coli* within the context of other studies (e.g. QS reporter strains) without any observable toxic effect on *E. coli*. Moreover, there are plasmid-borne QSSM synthase homologues which have been shown to be transferable between bacteria, and presumably this event would impose a fitness burden^48^,^49^. Since *E. coli* and *P. aeruginosa* have similar metabolic pathways involved in the production of QSSMs (Supplementary Fig. S1), it would also be predicted that any metabolic trade-off^7^ associated with QS signaling would be manifest similarly, and thus costs would have comparable impacts.

Although *E. coli* contains a protein capable of responding to the production of AHLs (SdiA)^50^,^51^,^52^,^53^, there is no AHL synthase, leading to the notion that *E. coli* can respond to an AHL cue from another species. It is not clear what SdiA regulates in *E. coli*, but it influences cell division, antibiotic resistance and virulence factor production when overproduced. SdiA does not preferentially bind the cognate AHLs for LasI and RhlI. SdiA has been shown not to interfere with a complete Las QS signalling cassette reporter^54^,^55^, and although it can bind to the promoter of *rhlI*, it does so regardless of the presence of the cognate AHL^56^. Importantly, the AHLs detected in our experiments did not overlap significantly with the AHLs shown previously to interact and activate SdiA^53^,^57^ and the effect of AHL production upon growth of an *sdiA* mutant mirrored that of MG1655 (data not shown).

The extended AHL profile of the *E. coli* RhlI-producer was limited to AHLs comprising short acyl chains. In contrast, 9 different long chain AHLs were detected in addition to the cognate OC_12_-HSL in the spent culture supernatants of the LasI-producer, with the quantities of the 3-oxo-series dominating. Interestingly, despite the LasI protein being produced at a higher level, the total level of AHLs and the level of the cognate AHL synthesized by LasI were similar to those made by RhlI (Supplementary Figs S2, S4 and S5). The greater effect this had upon growth is likely to result in part from the need for the longer acyl chain on the AHLs made by LasI (12 carbons compared to 4 carbons on the cognate AHLs of LasI and RhlI respectively). This would require greater metabolic investment from fatty acid biosynthesis pathways and thereby invoke a greater fitness cost. Despite the similar overall AHL levels, which would be predicted to require similar levels of the other shared substrate (SAM) and thus other AMC-related metabolites, there was a more substantial drain on SAM and other AMC metabolites that led to a corresponding fall in AI-2 and rise in MTA levels which in turn could influence polyamine levels since MTA is a feed-back inhibitor of polyamine synthesis. The reason for this requires further investigation, but could reflect changes in metabolic flux through the AMC triggered by the relative demands on fatty acid metabolism which are linked via cysteine (which feeds into coenzyme A biosynthesis and also the AMC). It was not clear why the RhlI inactive mutant S103E did not migrate with the same mobility as all other RhlI proteins analysed, but this may reflect a difference in conformational structure that in turn may influence AHL production by this particular protein.

### Evolutionary implications

Estimation of the metabolic costs and its consequences for absolute fitness in mono-culture and relative fitness in co-culture showed that the levels of specific central pathway metabolites are altered, and that this has an impact on growth and thus fitness to survive in mixed populations. In the context of horizontal transfer of QS systems, this metabolic perturbation was demonstrated to affect the levels of a native QSSM from the recipient cell, suggesting the potential for knock-on effects on the social environment.

Previous work has shown that the major cost to QS is the response to signal^3^. Until now, no one has experimentally isolated and investigated the source of costs underlying the production of QS signals that as public goods could evoke the display of trade-off strategies in deficient mutants^7^. Costly signal production could also influence social evolution as it could be subject to social cheating^11^. Self-interest can lead to a breakdown of cooperation at the group level (known as t this has an impact on growth and thus fitness to survive in mixed populaimal and human populations^36^). In environments where QS is important, social cheating in signal production could have important consequences^58^. In an infection scenario, it could affect the production of QS-controlled virulence factors. Signal cheating could thus lead to a loss in the ability of bacteria to infect their target hosts^40^.

The ability of the RhlI-producer to achieve the same final population density as the non-producer may reflect a delay in the collective benefit of QS. The production of QS-regulated public goods does not instantly exceed the cost of QSSM production. Once the population density reaches a threshold, the associated high level of QSSMs may provide a greater benefit in the context of QS regulated public goods production^27^. Interestingly, measurement of population density by OD in Fig. 1 indicated an approximate 10-fold fitness advantage in monoculture compared to the approximate 4-fold change in relative fitness calculated for Fig. 4 between non-producer and LasI-producer using viable cells (CFU). It was notable that in mixed culture (Fig. 4), the faster growth of the non-producer did not exhaust the overall nutrient supply and thereby greatly reduce the fitness of the LasI-producer in mixed culture versus monoculture. It is not clear what the underlying reason for this is, nor why the LasI-producer is fitter than the control in the un-induced mixed culture compared to the un-induced monoculture (Fig. 4) given that *E. coli* is incapable of responding to AHL signals, i.e. it cannot participate in a compensatory production of beneficial public goods. This observation suggests that the LasI-producer benefits from the presence of the other strain. There is a broad spread of relative fitness estimates for the uninduced mixed culture over the 4 parallel samples which may be reduced through inclusion of further replicas, but it is also possible that the control strain reaches stationary phase quickly, and some of the population lyses releasing nutrients that the LasI-producer can use to grow on, leading to a rise in viable cell numbers. Whatever the underlying reason, this finding accentuates the main finding of our experiments: that producing AHLs is costly. It would be interesting to extend this approach by determining the relative fitness of the different AHL-producing mutants that we generated, and also to determine whether RhlI-producers (that begin growing slowly but ultimately reach densities equal to non-producers in monoculture) are disadvantaged in mixed populations. Extending these studies would also facilitate measuring rates of ATP/signal turnover per cell during growth to enable calculations of the energetic cost of signal production to relate to theoretical values.

### Potential application

The correspondence between the higher level of LasI production and a more dramatic effect on growth and metabolite levels, offers the potential to experimentally titrate fitness costs by manipulating levels of AHL synthase production. This enhances the potential utility of bacterial QS as a model for testing aspects of signalling theory^1^. Furthermore, because QS contributes to bacterial virulence, our observation that AHL production perturbs the levels of central metabolites and has a knock-on effect on cell fitness provides impetus to drive the development of novel medical intervention strategies that target QS pathways.

## Methods

### Bacterial strains and growth conditions

Strains and plasmids used in this study (Supplementary Table S1) were routinely grown in Luria-Bertani medium (LB) or on nutrient agar plates at 37 °C. A MOPS minimal medium (MMM) was prepared as described previously^25^. Antibiotics were added at the following concentrations: carbenicillin 25 μg/ml; tetracycline 25 μg/ml and 100 μg/ml kanamycin. Isopropylthio-β-D-galactoside (IPTG) was added at a final concentration of 1 mM, unless otherwise indicated. Growth was followed by estimating optical densities at 600 nm using a 1 in 10 dilution of cultures into the growth medium to ensure accurate spectrophotometer readings, or using viable cell counts (colony forming units: CFU).

### DNA manipulation and cloning procedures

DNA was purified using a plasmid purification kit (Qiagen) or Wizard genomic DNA purification kit (Promega). Restriction enzyme digestion, ligation and agarose gel electrophoresis were performed using standard methods^59^. Restriction fragments were routinely purified from agarose gels using a QIAquick kit (Qiagen). Transformation of *E. coli* was carried out by electroporation^60^. Oligonucleotide primers (Supplementary Table S1) were synthesised by Sigma Genosys. Both strands of cloned PCR products were sequenced by the DNA Sequencing Laboratory at the University of Nottingham (United Kingdom). Nucleotide and deduced amino acid sequences were aligned using Clustal W (http://clustalw.genome.jp/).

### Plasmid construction and site directed mutagenesis

The 606-bp *lasI* and *rhlI* genes were PCR-amplified using chromosomal DNA of *P. aeruginosa* PA01 as the template and the primer pairs *lasI* Forward/*lasI* Reverse or *rhlI* Forward/*rhlI* Reverse respectively. The purified 0.624 kb fragments were ligated into the pGEM-T Easy vector (designated pGEMT-*lasI* or pGEMT-*rhlI*) and released with *Eco*RI-*St*uI digestion for cloning into shuttle vector pME6032 (pME-*lasI* and pME-*rhlI*).

To mutate the *lasI* and *rhlI* genes, degenerated phosphorylated PCR primer pairs amplified pGEMT-*lasI* or pGEMT-*rhlI*. Following recircularisation and transformation into DH5α, the *lasI* and *rhlI* mutant fragments were recovered from pGEMT-Easy and inserted as *Eco*RI-*St*uI fragments into the pME6032 vector. The point mutations were confirmed by sequencing.

SDS-PAGE and western blotting. This was undertaken as described previously^61^. Anti-RhlI was diluted to 1:2000 and anti-mouse IgG HRP (Sigma) to 1:1000.

### Small molecule analysis

AHLs were quantified by LC-MS/MS as described previously^44^. For extraction of intracellular AMC metabolites, bacteria were grown in 125 ml IPTG supplemented LB in 500 ml Erlenmeyer flasks at 37 °C for 12 h. Samples of 5 ml containing equivalent cell densities for each strain were quenched with 15 ml of phosphate-buffered saline (PBS), cells were lysed, metabolites derivatized and detected^22^. To determine intracellular MTA levels, the existing protocol^22^ was modified to use 100% MeOH for extraction and samples were reconstituted in 100 μl of dH_2_0 and analysed by liquid chromatography-tandem mass spectrometry using a Quattro Ultima (Waters Micromass, Manchester, UK) in conjunction with an Agilent 1100 LC system (Agilent Technologies, Waldbron, Germany) with a cooled autosampler [30]. HPLC was carried out using a Synergi 4u Hydro-RP (4 μm, 150_ 2.0 mm, Phenomenex, Macclesfield, UK) with a guard column fitted. AI-2 was quantified by^24^ and expressed as the change in bioluminescence of the reporter strains (bioluminescence in the presence of extract/background bioluminescence in the presence of sterile medium).

### Mixed Population Competition Assay

Approximately 10^5^ cells from overnight cultures of each strain or a 1:1 mixture of two strains were inoculated into 2 ml MMM + tetracycline. Four wells of each population were supplemented with IPTG and four left uninduced. Cultures were grown shaking for 24 h at 37 °C. An aliquot of each population was diluted and plated onto two LB + tetracycline agar plates for colony counting. To calculate the final proportion of each strain in the mixed populations, 50 colonies from each mixed population were randomly selected and streaked across an aliquot of the *E coli* biosensor pSB1075^62^ on LB + tetracycline + IPTG agar plates. The biosensor carries a *lux* reporter that is expressed in the presence of OC_12_-HSL, thus cross-streaking with *E. coli* MG1655(pME-*lasI*) produces luminescent streaks after overnight growth. A Hamamatsu Aequoria darkbox and M4314 Image Intensifier Controller were used along with the software Wasabi 1.5 to image plates and score numbers of light and dark streaks. To calculate the false negative and false positive rates, ten colonies from each pure population were also streaked across the biosensor and imaged after overnight growth. Bayes’ theorem was applied to these data to calculate the probability that a light streak was *E. coli* MG1655(pME-*lasI*) and that a dark streak was *E. coli* MG1655(pME6032); these were 0.99 and 0.94 respectively and we adjusted observed light/dark numbers in the mixed populations to take account of this.

The evolutionary fitness of *E. coli* MG1655(pME-*lasI*) relative to *E. coli* MG1655(pME6032) in mixed populations was calculated using Equation (1).
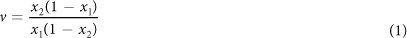
where *x*_1_ and *x*_2_ are the initial and final frequencies of *E. coli* MG1655(pME-*lasI*) in the population, respectively^62^. When the two genotypes have equal fitness, *x*_1_ = *x*_2_ and *v* = 1. Values of *v* < 1 reflect being outcompeted by MG1655(pME6032) and values >1 indicate that MG1655(pME-*lasI*) outcompetes MG1655(pME6032). The relative fitness of *E. coli* MG1655(pME-*lasI*) in pure culture was calculated by randomly pairing pure MG1655(pME-*lasI*) and MG1655(pME6032) populations with IPTG treatment and applying the same formula. Data were analysed using ANOVA in R 2.14.0^63^. Total population size and relative fitness were both square root transformed to meet the assumptions of parametric tests and when dropping of an outlier in the growth data caused loss of orthogonality the *car* package^64^ was used to implement ANOVA with Type II sums of squares^65^,^66^.

## Additional Information

**How to cite this article**: Ruparell, A. *et al*. The fitness burden imposed by synthesising quorum sensing signals. *Sci. Rep.* **6**, 33101; doi: 10.1038/srep33101 (2016).

## Supplementary Material

Supplementary Information
