# Supplementary material for: The fitness burden imposed by synthesising quorum sensing signals
Source: Sci Rep. 2016 Sep 12;6:33101. doi: 10.1038/srep33101 (PMC5018880; doi:10.1038/srep33101)
Supplement: Supplementary Information [file srep33101-s1.pdf]

Supplementary Information:

The fitness burden imposed by synthesising quorum sensing signals.

Ruparell A.,[1#] Dubern JF.,[1] Ortori CA.,[2] Harrison, F. [1], Halliday NM.,[1] Emtage A.,[2β] Ashawesh, MM., [1] Laughton CA.,[2] Diggle, SP.[1], Williams P., [1] Barrett DA.,[2] and Hardie KR.[1*]

School of Life Sciences, Centre for Biomolecular Sciences, University of Nottingham, University Park, Nottingham, NG7 2RD, United Kingdom[1]

Centre for Analytical Bioscience, School of Pharmacy, University of Nottingham, Nottingham NG7 2RD, United Kingdom[2]

Current Address: Mars Waltham Centre for Pet Nutrition, United Kingdom[#]

Current Address: University of Nottingham, Malaysia [β]

*Corresponding author:

Dr Kim Rachael Hardie, School of Life Sciences, Centre for Biomolecular Sciences, University of Nottingham, University Park, Nottingham, NG7 2RD, Tel: 0115 8467958, Fax: 0115 8467951, Email: kim.hardie@nottingham.ac.uk

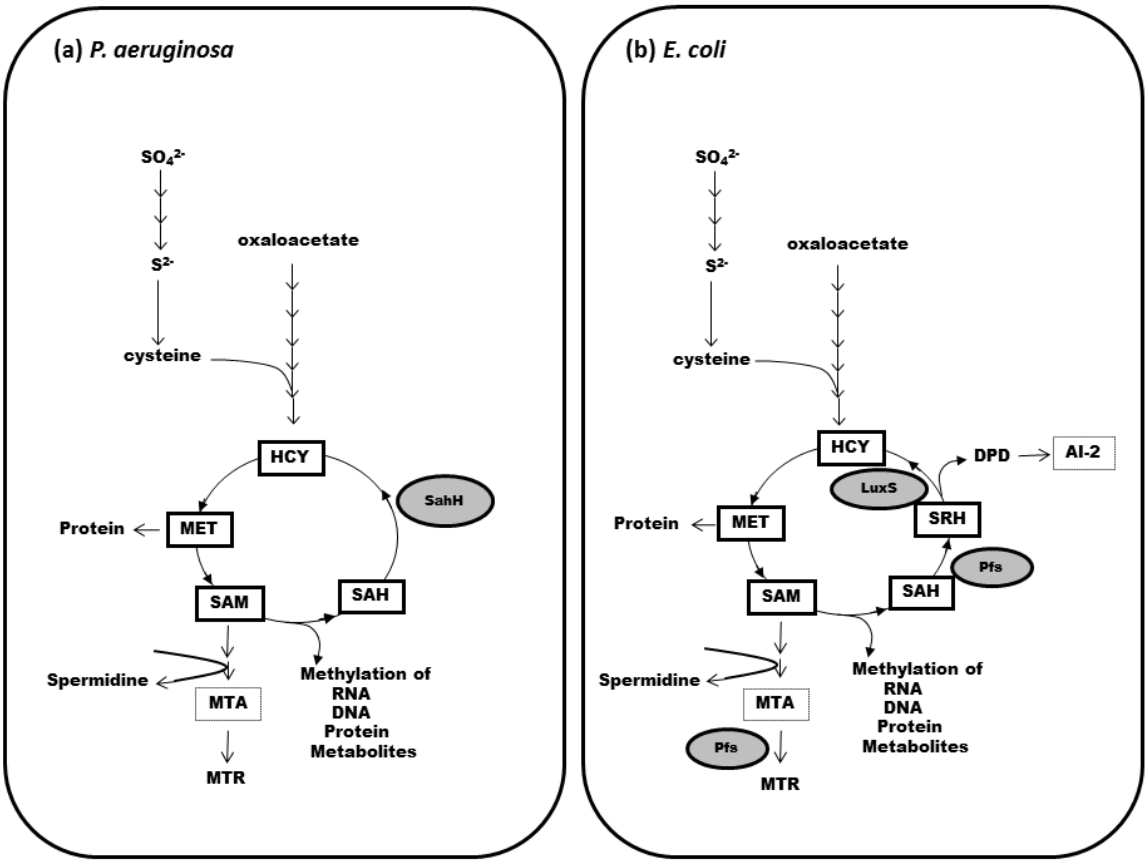

Supplementary Figure S1. The activated methyl cycle of (a) *P. aeruginosa* and (b) *E. coli*. In *P. aeruginosa* SAH hydrolase (SahH) converts SAH (*S*-adenosyl homoserine) to HCY (homocysteine) directly, but in *E. coli* the enzymes Pfs and LuxS work sequentially generating the intermediate SRH (*S*-ribosyl homoserine) and the additional product DPD (4,5-dihydroxy-2,3-pentanedione) which is converted to AI-2 (autoinducer 2). In *E. coli*, Pfs has a secondary role in conversion of MTA (5'-methylthioadenosine) to MTR (5'-methylthioribose). MET: methionine, SAM: *S*-adenosyl-L-methionine.

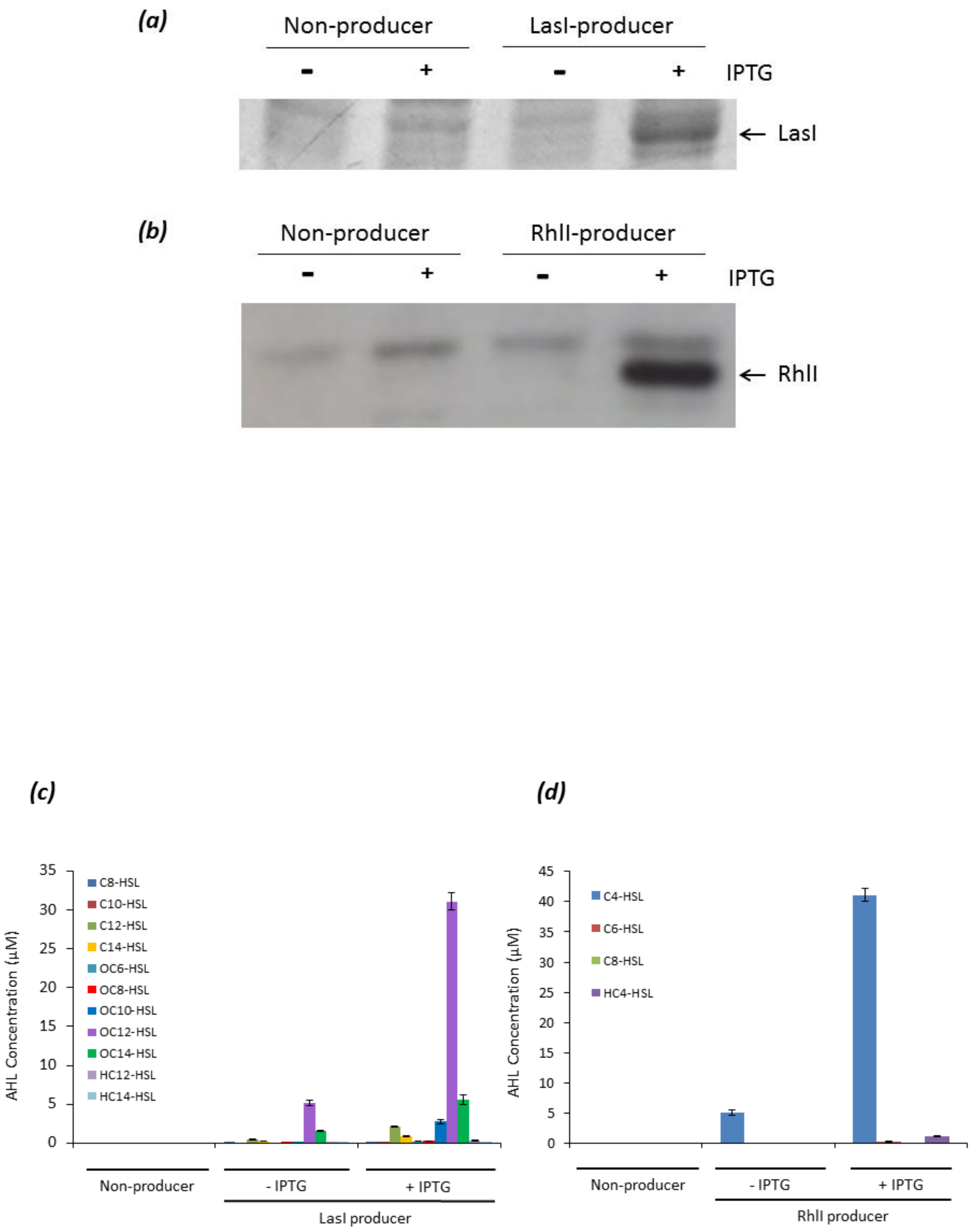
(a)
Non-producer
LasI-producer
- + - + IPTG
← LasI
(b)
Non-producer
RhlI-producer
- + - + IPTG
← RhlI
(c)
AHL Concentration (µM)
35
30
25
20
15
10
5
0
C8-HSL
C10-HSL
C12-HSL
C14-HSL
OC6-HSL
OC8-HSL
OC10-HSL
OC12-HSL
OC14-HSL
HC12-HSL
HC14-HSL
Non-producer
- IPTG
+ IPTG
LasI producer
(d)
AHL Concentration (µM)
45
40
35
30
25
20
15
10
5
0
C4-HSL
C6-HSL
C8-HSL
HC4-HSL
Non-producer
- IPTG
+ IPTG
RhlI producer

Supplementary Figure S2. AHL synthases direct the synthesis of their cognate QSSMs in a heterologous host. *E. coli* strains MG1655[pME6032], MG1655[pME-*rhlI*] and MG1655[pME-*lasI*] were grown in LB + tetracycline until an OD_{600} = 0.5 and induced with 1 mM IPTG for 2 h. Whole cell extracts were separated by SDS PAGE and the presence of LasI **(panel *a*)** was detected by Coomassie staining whist the lower levels of RhlI were detected by Immunoblotting with a specific antisera **(panel *b*)**. Quantitative profiling of AHLs produced by the same strains was undertaken by extracting with acidified ethyl acetate from LB or late exponential phase supernatants of *E. coli* strains MG1655[pME6032] (OD_{600} = 0.8), MG1655[pME-*lasI*] (OD_{600} = 0.9), and MG1655[pME-*rhlI*] (OD_{600} = 0.9) grown in LB containing IPTG. The actual concentration (µM) determined by LC-MS/MS analysis of AHLs described in Experimental Procedures for *lasI* **(c)** and *rhlI* **(*d*)** is shown. Of the 11 AHLs detected in cultures harvested from *lasI* induced cells OC_{10}-HSL, OC_{12}-HSL and OC_{14}-HSL were the dominant signals. Other AHLs present below 5 µM were C_8-HSL, C_{10}-HSL and C_{12}-HSL, C_{14}-HSL, OC_6-HSL, OC_8-HSL, HC_{12}-HSL and HC_{14}-HSL. The 4 AHLs detected in culture supernatant of MG1655[pME-*rhlI*] were C_4-HSL, C_6-HSL, C_8-HSL and HC_4-HSL. The data are means ± standard deviations for three independent extractions.

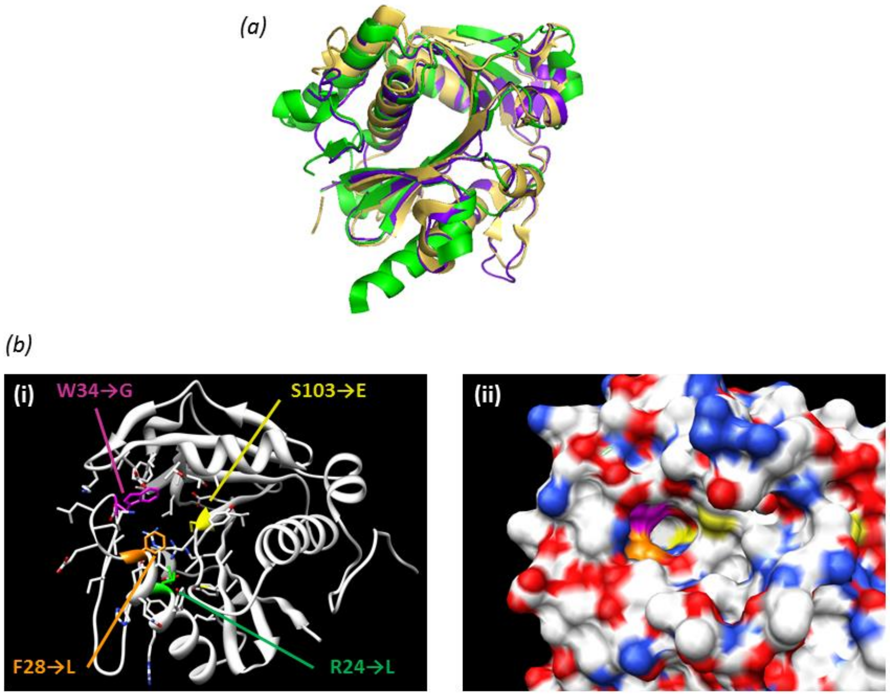

Supplementary Figure S3. RhlI modelling to inform the creation of catalytically inactive QSSM synthase mutants. *P. aeruginosa* RhlI model (purple) was predicted using multi-template homology modelling, and utilised the structures of *P. aeruginosa* LasI (yellow) and *P. stewartii* EsaI (green) **(*a*)**. The positions of the LasI and RhlI conserved residues selected for mutation in this study are shown on the ribbon **(*b*)** and space filling **(*c*)** models on the predicted structure of RhlI. Colours indicate R23 (green), F28 (orange), W34 (pink) and S103W (yellow). **RhlI Homology Modelling.** An atomised model of RhlI was constructed using MODELLER9v7 [31] and the homologs *P. aeruginosa* LasI (30% identity with RhlI sequence; pdb code 1r05) and *P. stewartii* EsaI (22% identity with RhlI sequence; pdb code 1kzf) as templates. The model with the lowest DOPE score [32] was chosen as the best model and used for visualization of the RhlI protein. The model was checked and hydrogen atoms added/refined using MOLPROBITY [33]. The resulting ramachandran plot demonstrated that 92.5% of model residues were in favoured regions.

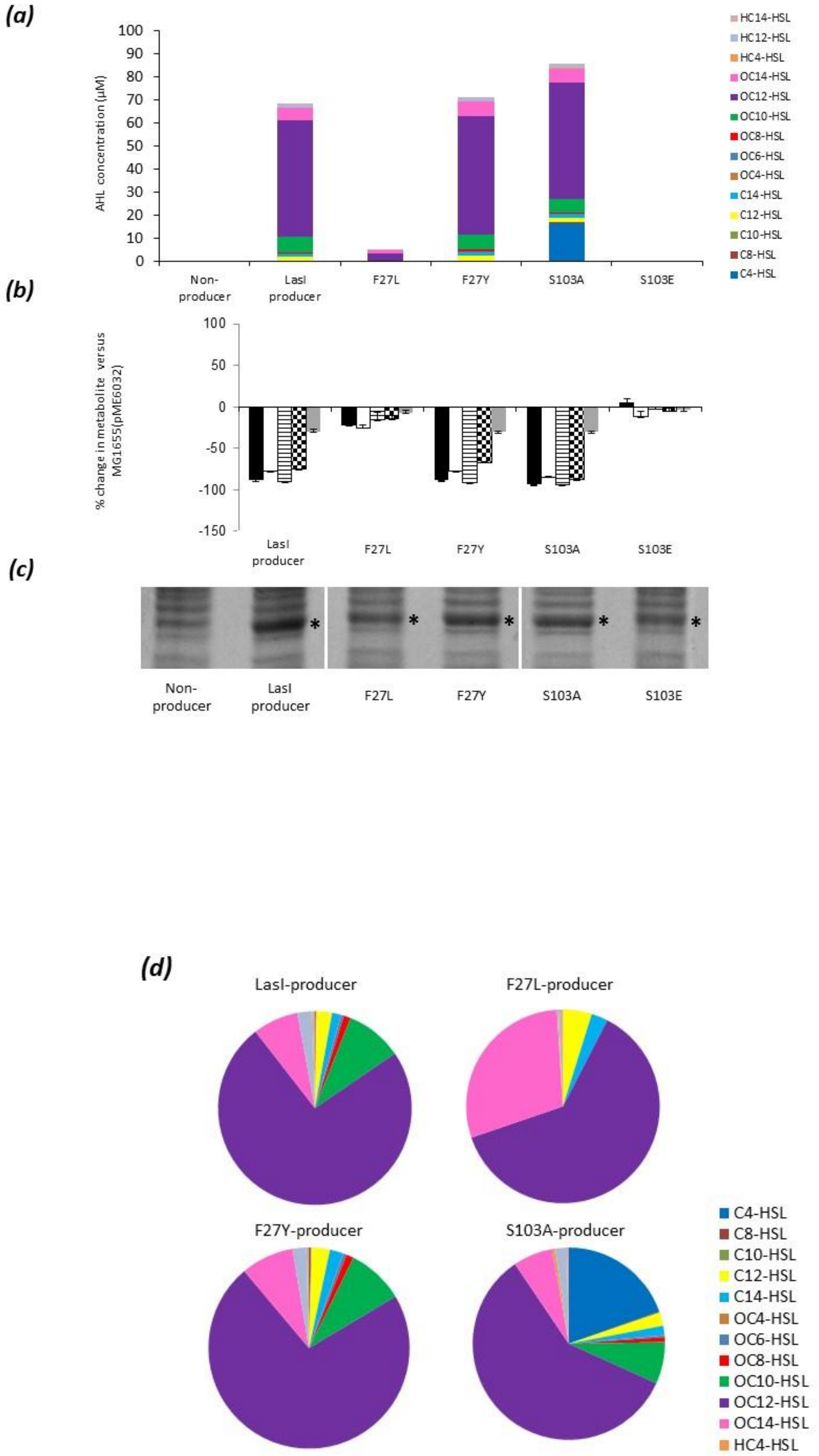

(a)
AHL concentration (µM)
HC14-HSL
HC12-HSL
HC4-HSL
OC14-HSL
OC12-HSL
OC10-HSL
OC8-HSL
OC6-HSL
OC4-HSL
C14-HSL
C12-HSL
C10-HSL
C8-HSL
C4-HSL
Non-producer
LasI producer
F27L
F27Y
S103A
S103E
(b)
% change in metabolite versus MG1655(pME6032)
LasI producer
F27L
F27Y
S103A
S103E
(c)
Non-producer
LasI producer
F27L
F27Y
S103A
S103E
(d)
LasI-producer
F27L-producer
F27Y-producer
S103A-producer
C4-HSL
C8-HSL
C10-HSL
C12-HSL
C14-HSL
OC4-HSL
OC6-HSL
OC8-HSL
OC10-HSL
OC12-HSL
OC14-HSL
HC4-HSL

(e) **Rich medium**

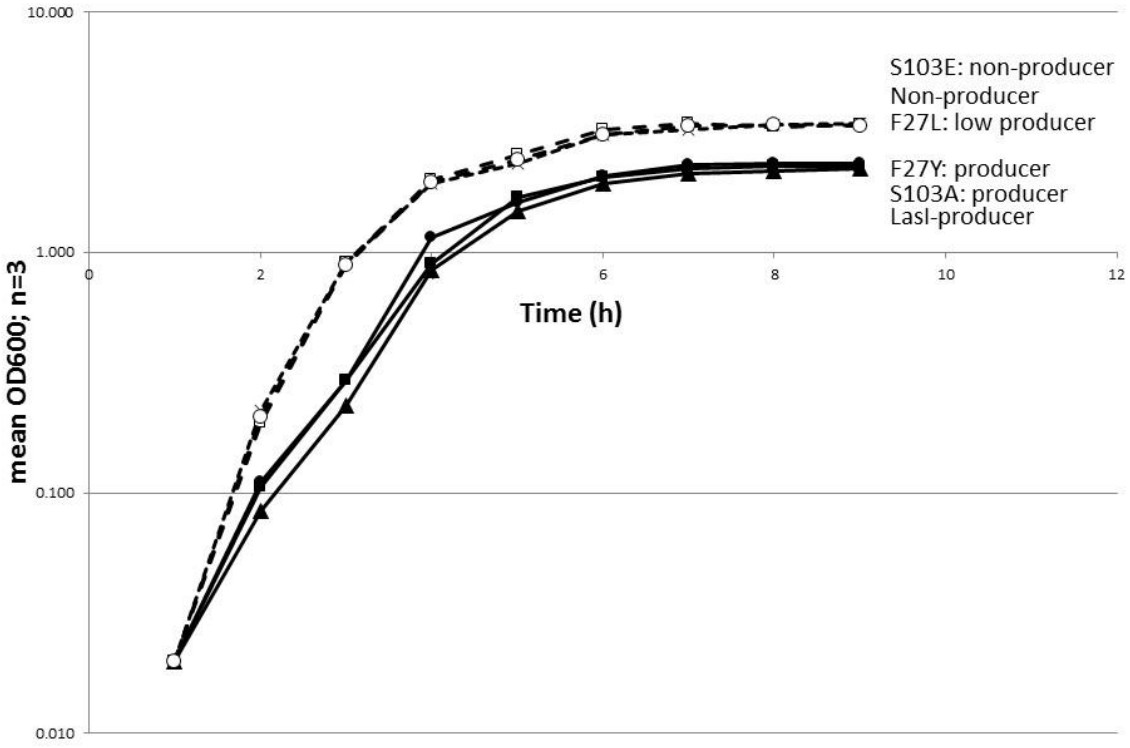

(f) **Minimal medium**

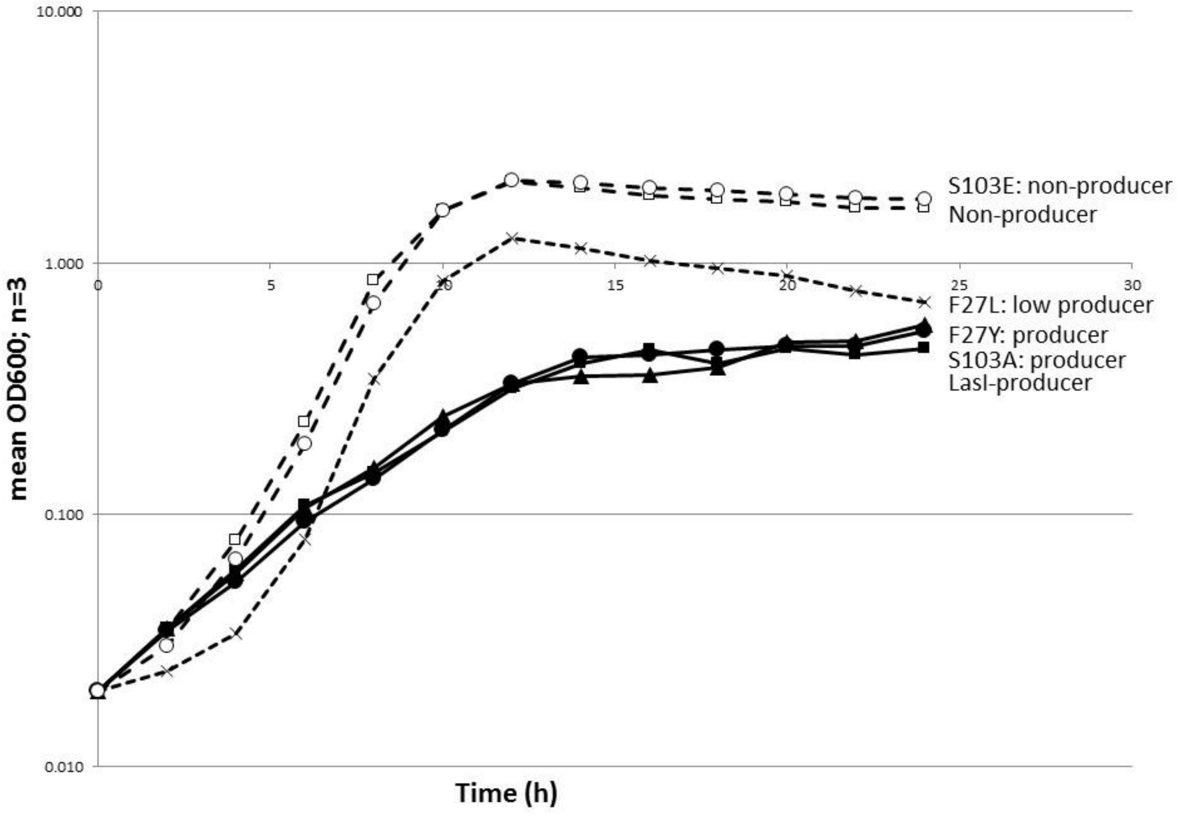

Supplementary Figure S4. QSSM synthase mutants lacking the ability to generate AHLs no longer impose a fitness cost and fail to make an impact on the levels of AMC metabolites: LasI. *E. coli* MG1655 bearing the empty vector (pME6032) or a derivative encoding WT LasI (pME-*lasI*), or LasI mutated to introduce the change F27L, F27Y, S103A, or S103E, were grown in the presence or absence of IPTG as indicated. The production of LasI was monitored by Coomassie staining the SDS PAGE (**panel (*c*)**, marked with an asterix). Size was estimated by comparison with the molecular weight markers. **Panel (*a*)** shows the AHLs extracted from late exponential phase supernatants of each strain with acidified ethyl acetate and profiled by LC-MS/MS analysis as described in Experimental Procedures. The ratio between the different signalling molecules present in supernatants is represented by pie charts in **Panel (*d*).** The number of repeats represented is 3. **Panel (*b*)** Metabolite levels were determined in the same strains following growth in LB with IPTG induction until OD 0.8-0.9. Intracellular accumulation of SAM (black), SAH (white), SRH (horizontal stripes), HCY (checked) and MET (grey) was determined by LC-MS analysis. The peak area corresponding to each compound in an extract was divided by the peak area of an appropriate internal standard (IS) for normalisation; the data are the means ± standard deviations for three independent cultures. The same *E. coli* SDM strains were inoculated into 125 ml LB + tetracycline media **Panel (*e*)** or MMM + tetracycline **Panel (*f*)** and grown shaking at 37°C in 500 ml-Erlenmeyer flasks. Aliquots (1 ml) were taken at regular intervals as indicated, and the mean OD_{600} values of triplicate culture samples are shown on a $\log_{10}$ scale over time (h). Error bars indicate standard deviations from the means. Strains generating wild type levels of AHLs are indicated by the solid lines and closed symbols (pME-*lasI*, *lasI*S103A, *lasI*F27Y), those not making detectable AHLs by the dashed lines and open symbols (pME6032, *lasI*F27L,

*lasI*S103E), and those producing intermediate levels by the dotted lines with cross symbols (MG1655(pME-*lasI*F27L)). The MG16 (pME6032) negative control is indicated by open squares and the positive control by closed squares: MG1655(pME-*las*I). The symbols used in the figure are closed triangles for the *lasI*F27Y, closed circles for *lasI*S103A, open circles for *lasI*S103E.

(a)

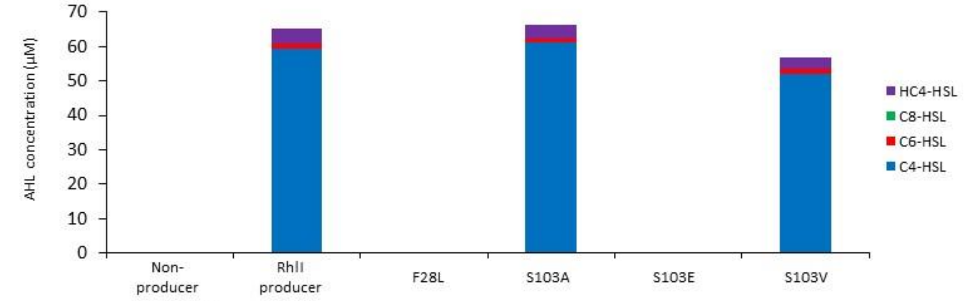

AHL concentration (µM)
70
60
50
40
30
20
10
0
Non-producer
RhlI producer
F28L
S103A
S103E
S103V
HC4-HSL
C8-HSL
C6-HSL
C4-HSL

(b)

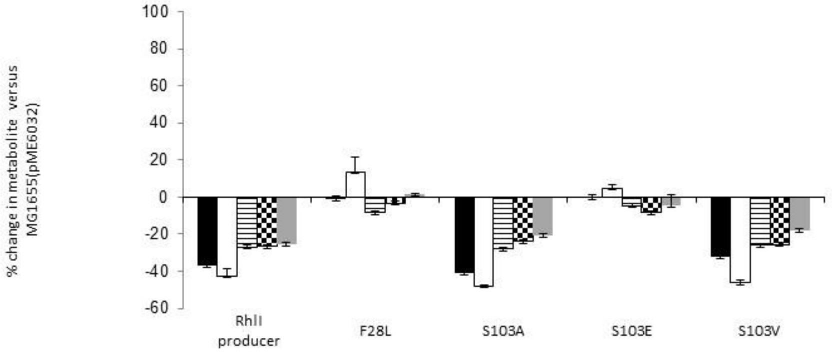

% change in metabolite versus MG1655(pME6032)
100
80
60
40
20
0
-20
-40
-60
RhlI producer
F28L
S103A
S103E
S103V

(c)

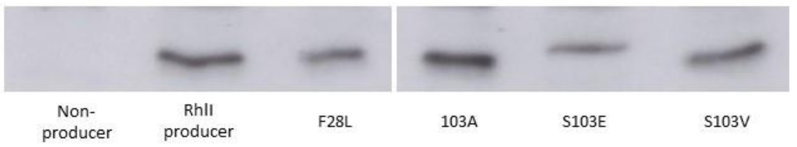

Non-producer
RhlI producer
F28L
103A
S103E
S103V

(d)

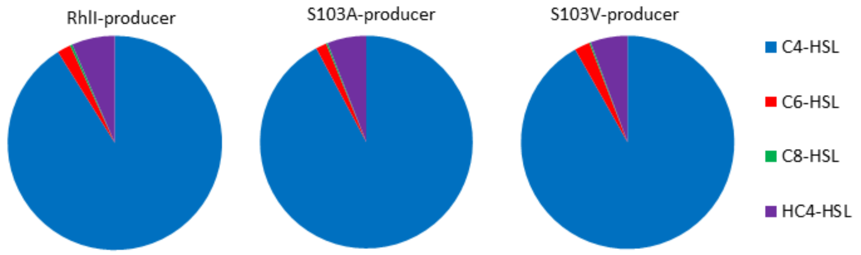

RhlI-producer
S103A-producer
S103V-producer
C4-HSL
C6-HSL
C8-HSL
HC4-HSL

(e) **Rich medium**

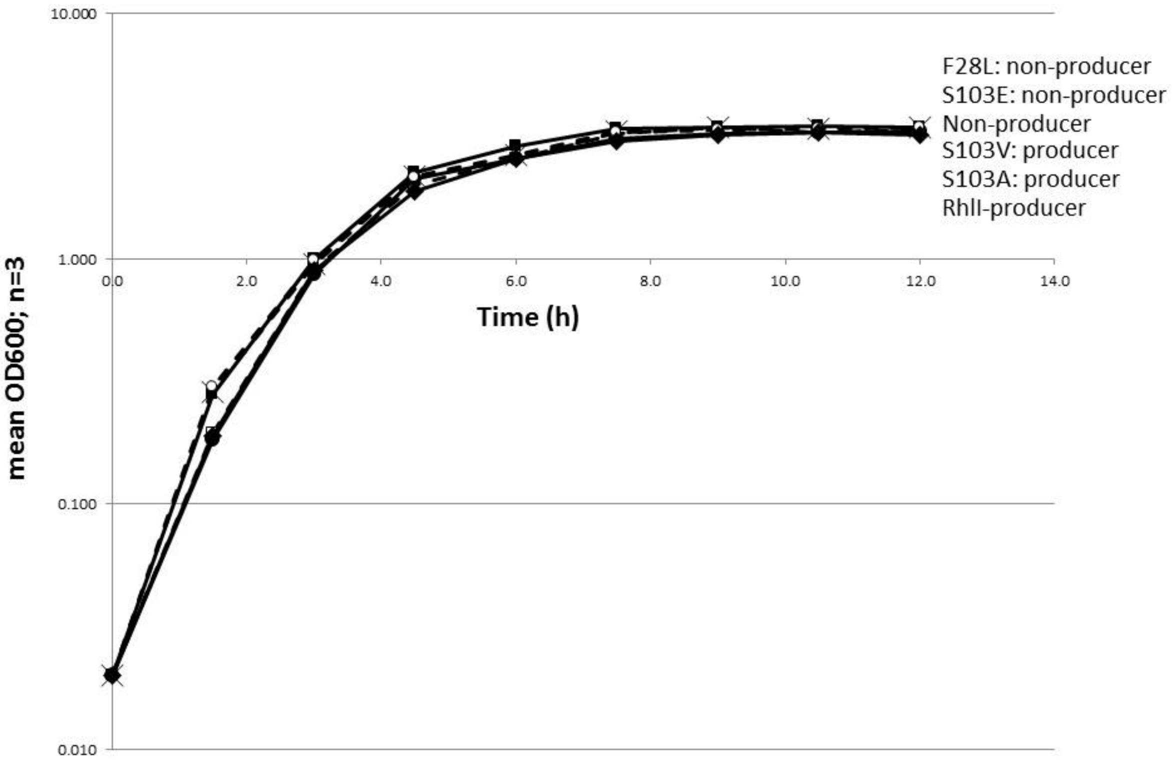

(f) **Minimal medium**

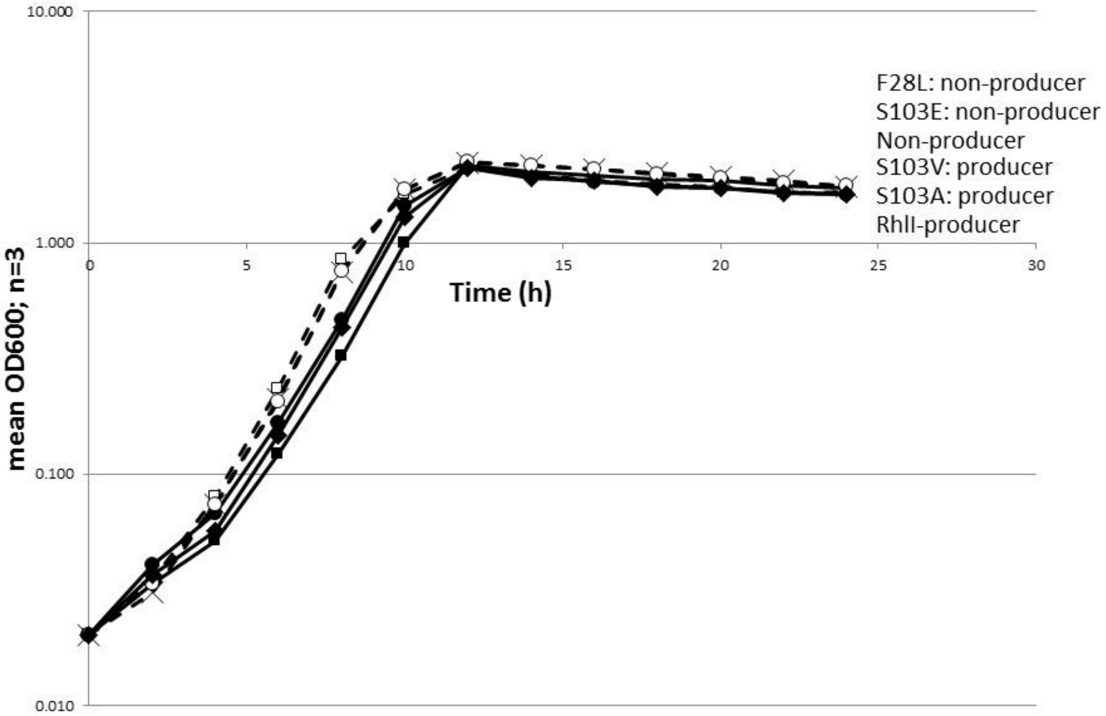

Supplementary Figure S5. QSSM synthase mutants lacking the ability to generate AHLs no longer impose a fitness cost and fail to make an impact on the levels of AMC metabolites: RhlI. *E. coli* MG1655 bearing the empty vector (pME6032) or a derivative encoding WT RhlI (pME-*rhlI*), or RhlI mutated to introduce the change F28L, S103A, S103E, or S103V were grown in the presence or absence of IPTG as indicated. The production of RhlI was monitored by immunoblotting with anti-RhlI (**panel (*c*)**). Size was estimated by comparison to the molecular weight markers. **Panel (*a*)** shows the AHLs extracted from late exponential phase supernatants of each strain with acidified ethyl acetate and profiled by LC-MS/MS analysis as described in Experimental Procedures. The ratio between the different signalling molecules present in supernatants is represented by pie charts in **Panel (*d*).** This was repeated 3 times. **Panel (*b*)** Metabolite levels were determined in the same strains following growth in LB + tetracycline with IPTG induction until OD 0.8-0.9. Intracellular accumulation of SAM (black), SAH (white), SRH (horizontal stripes), HCY (checked) and MET (grey) was determined by LC-MS analysis. The peak area corresponding to each compound in an extract was divided by the peak area of an appropriate internal standard (IS) for normalisation; the data are the means ± standard deviations for three independent cultures. The same *E. coli* SDM strains were inoculated into 125 ml LB + tetracycline media **Panel (*e*)** or MMM + tetracycline **Panel (*f*)** and grown shaking at 37°C in 500 ml-Erlenmeyer flasks. Aliquots (1 ml) were taken at regular intervals as indicated, and the mean OD_{600} values of triplicate culture samples are shown on a log_{10} scale over time (h). Error bars indicate standard deviations from the means. Strains generating wild type levels of AHLs are indicated by the solid lines and closed symbols (pME-*rhlI*, *rhlI*S103A, *rhlI*S103V), those not making detectable AHLs by the dashed lines and open symbols (pME6032, *rhlI*F27L,

*rhlI*S103E). The MG1655(pME6032) negative control is indicated by open squares and the positive control by closed squares: MG1655(pME-*rhlI*). The symbols used in the figure are closed diamonds for the *rhlI*S1-3V, closed circles for *rhlI*S103A, open circles for *rhlI*S103E.

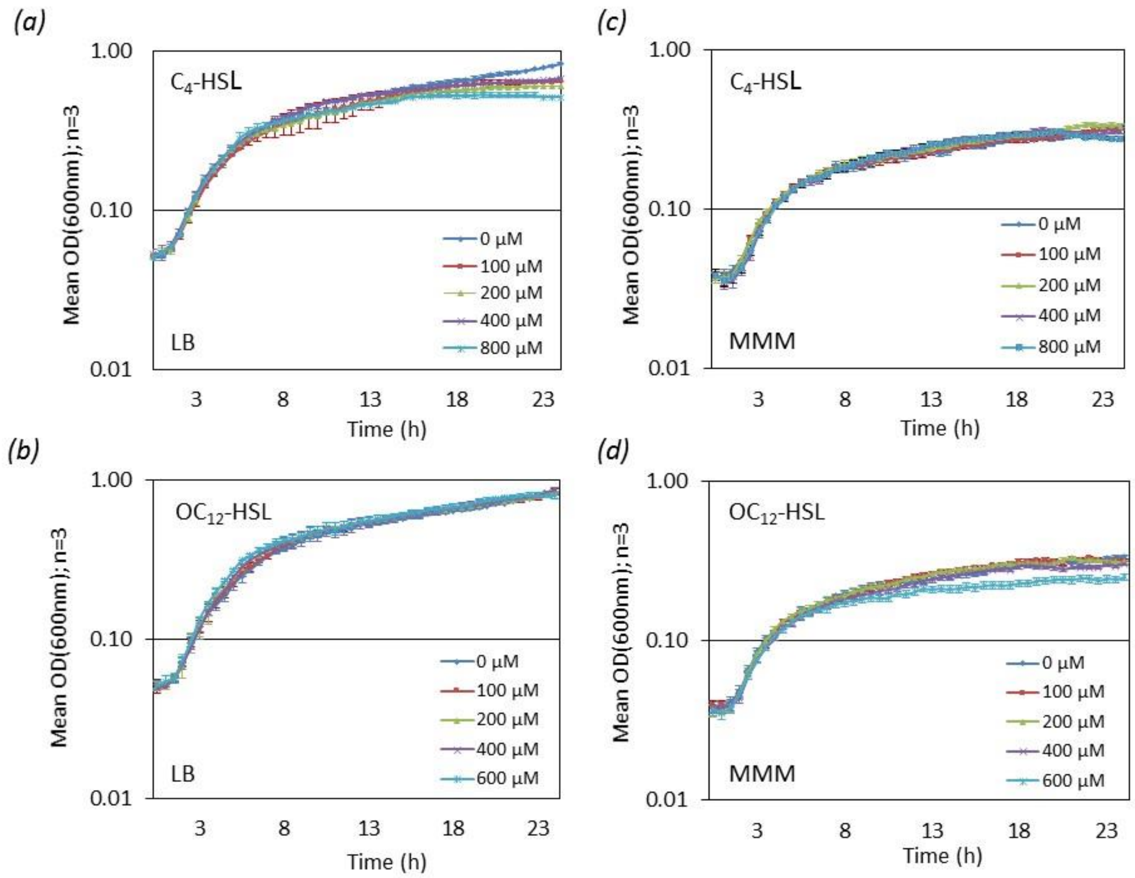

Supplementary Figure S6. Exogenous addition of QSSMs does not affect growth of a heterologous host. *E. coli* strain MG1655[pME6032] was grown with varying concentrations of C4-HSL or OC12-HSL added exogenously into LB (a, b) or MMM cultures (c, d) at the start of the experiment. The optical density was determined during growth every 30 min at wavelength of 600 nm using a TECAN microplate reader. Standard deviations are based on the mean values of three parallel cultures.

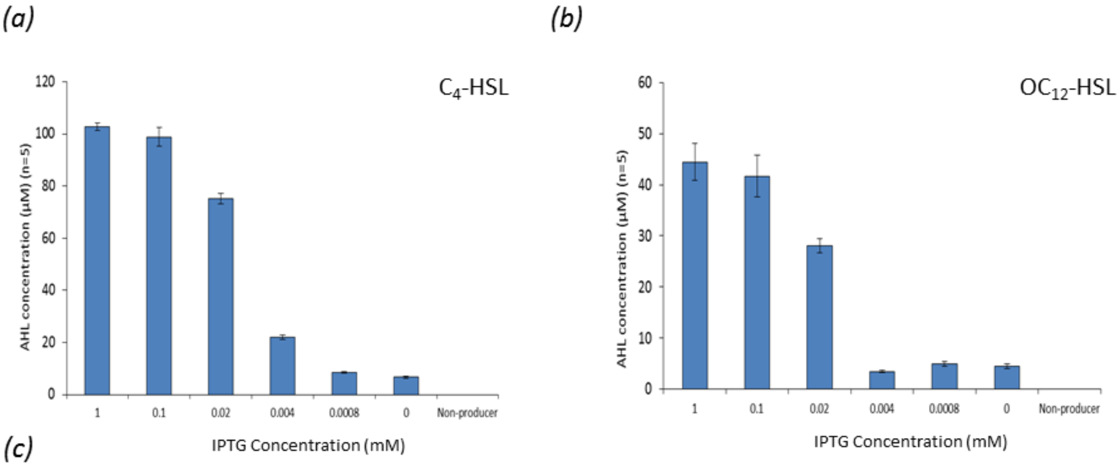

(c)

	Growth rate (h^{-1})							Population Density (OD600nm)					
IPTG (mM)	1	0.1	0.02	0.004	0.0008	0		1	0.1	0.02	0.004	0.0008	0
Non producer	0.48 ± 0.01	0.50 ± 0.01	0.48 ± 0.01	0.48 ± 0.02	0.49 ± 0.01	0.49 ± 0.01		1.32 ± 0.05	1.32 ± 0.04	1.35 ± 0.02	1.35 ± 0.09	1.12 ± 0.04	1.06 ± 0.01
LasI producer	0.27 ± 0.03***	0.38 ± 0.04*	0.50 ± 0.01	0.46 ± 0.01	0.49 ± 0.01	0.49 ± 0.02		0.47 ± 0.03***	0.48 ± 0.02***	0.65 ± 0.01***	1.10 ± 0.06	0.95 ± 0.03	0.98 ± 0.02
RhlI producer	0.44 ± 0.00	0.44 ± 0.00	0.44 ± 0.00	0.44 ± 0.01	0.44 ± 0.01	0.45 ± 0.01		0.84 ± 0.02***	0.88 ± 0.01***	0.97 ± 0.07***	1.02 ± 0.01	1.03 ± 0.02	1.06 ± 0.03

Supplementary Figure S7. Limiting QSSM production by reducing IPTG induction of the synthase repairs growth defects. Varying concentrations of IPTG were added exogenously into the LB culture at the start of the experiment. The level of OC12-HSL synthesised by a LasI-producer (panel a) and C4-HSL synthesised by a RhlI-producer (panel b) was determined in stationary phase cultures by LC-MS/MS, as described in material and methods. The optical density was determined during growth in LB medium every 30 min at wavelength of 600nm using a TECAN microplate reader. Growth rate and optical density reached at stationary phase for *E. coli* MG1655 non-producer, LasI-producer and RhlI-producer are shown in panel c. Standard deviations are based on the mean values of five replicates for the quantification of AHLs and three replicates for the determination of growth rate and optical density at stationary. Statistical significance between producer and non-producer was determined using a unpaired *t*-test (* P<0.05; ** P<0.01; ***P<0.001).

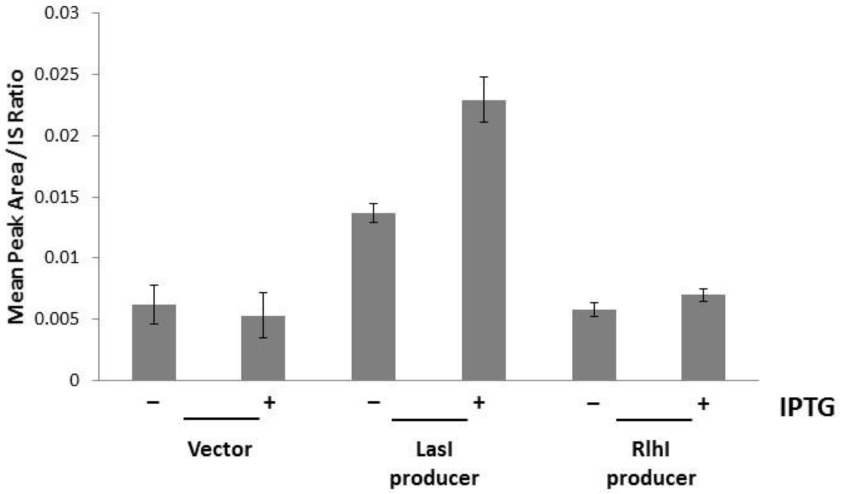

Supplementary Figure S8. AHL synthase LasI increases intracellular MTA levels in WT *E. coli* MG1655. Intracellular accumulation of MTA was determined by LC-MS analysis in *E. coli* strains MG1655 (pME6032), MG1655 (pME-*lasI*) and MG1655 (pME-*rhl)* grown in LB + tetracyline with and without IPTG. Peak area corresponding to each compound was divided by the peak area of the appropriate internal standard (IS) for normalisation. Metabolite levels for a single experiment are shown.

Supplementary References

1] Sali, A. & Blundell, T. L. Comparative protein modelling by satisfaction of spatial restraints. *J Mol Biol* **234**, 779-815 (1993).

[2] Shen, M. Y. & Sali, A. Statistical potential for assessment and prediction of protein structures. *Protein Sci* **15**, 2507-2524 (2006).

[3] Davis, I. W., Leaver-Fay, A., Chen, V. B., Block, J. N., Kapral, G. J., Wang, X., Murray, L. W., Arendall, W. B.,3rd, Snoeyink, J., Richardson, J. S. & Richardson, D. C. MolProbity: all-atom contacts and structure validation for proteins and nucleic acids. *Nucleic Acids Res* **35**, W375-83 (2007).

Supplementary Table S1. Strains, plasmids and primers used in this study.

Strain, plasmid or oligonucleotide	Description and/or sequence (5'-3')[a]	Reference or source
Strains		
Escherichia coli		
DH5α	F⁻ *endA1 hsdR17 supE44 thi-1 recA1 gyrA96 relA1* Δ(*lacZYA-argF*)U169 *deoR* Δ(ϕ80d*lacZ*ΔM15)	(1)
MG1655	*F⁻ λ⁻*	(2)
MG1655Δ*pfs*	*pfs'::*Km[r] derivative of MG1655	(3)
JM109	F9 *traD36 lacI*q D(*lacZ*)M15 *proA*1*B*1/*el4*2 D(*lac-proAB*) *thi gyrA96 endA1 hsdR17 relA1 supE44 recA1*	(4)
Vibrio harveyi		
BB170	Biosensor AI-1⁻, biosensor AI-2⁺	(5)
Plasmids		
pGEM-T Easy	Cloning vector, Cb[r], Amp[r], *lacZ*, allowing selection via blue-white screening	Promega
pME6032	Cloning vector for overexpression under the IPTG-inducible *tac* promoter, Tc[r]	(6)
pME-*lasI*	pME6032 derivative carrying the *lasI* gene under P*tac* control, Tc[r]	This study
pME-*lasI* R23W	pME-*lasI* derivative carrying a substitution of Arg for Try at codon 23 of the *lasI* ORF	This study
pME-*lasI* F27L	pME-*lasI* derivative carrying a substitution of Phe for Leu at codon 27 of the *lasI* ORF	This study
pME-*lasI* F27Y	pME-*lasI* derivative carrying a substitution of Phe for Tyr at codon 27 of the *lasI* ORF	This study
pME-*lasI*: W33G	pME-*lasI* derivative carrying a substitution of Try for Gly at codon 33 of the *lasI* ORF	This study
pME-*lasI* S103A	pME-*lasI* derivative carrying a substitution of Ser for Ala at codon 103 of the *lasI* ORF	This study
pME-*lasI* S103E	pME-*lasI* derivative carrying a substitution of Ser for Glu at codon 103 of the *lasI* ORF	This study
pME-*lasI* S103V	pME-*lasI* derivative carrying a substitution of Ser for Val at codon 103 of the *lasI* ORF	This study
pME-*rhlI*	pME6032 derivative carrying the *rhlI* gene under P*tac* control, Tc[r]	This study
pME-*rhlI* F28L	pME-*rhlI* derivative carrying a substitution of Phe for Leu at codon 28 of the *rhlI* ORF	This study
pME-*rhlI* F28Y	pME-*rhlI* derivative carrying a substitution of Phe for Tyr at codon 28 of the *rhlI* ORF	This study
pME-*rhlI* W34G	pME-*rhlI* derivative carrying a substitution of Try for Gly at codon 34 of the *rhlI* ORF	This study
pME-*rhlI* S103A	pME-*rhlI* derivative carrying a substitution of Ser for Ala at codon 103 of the *rhlI* ORF	This study
pME-*rhlI* S103E	pME-*rhlI* derivative carrying a substitution of Ser for Glu at codon 103 of the *rhlI* ORF	This study
pME-*rhlI* S103V	pME-*rhlI* derivative carrying a substitution of Ser for Val at codon 103 of the *rhlI* ORF	This study
pSB1142	*lux*-based acyl-HSL bioreporter, Tc[r]	(7)
pSB536	*lux*-based acyl-HSL bioreporter, Amp[r]	(7)
Primers		

lasI-F	TATCAATTG**ATG**ATCGTACAAATTGGTCGG, with an underlined *Mfe*I restriction site directly upstream of the start codon (bold) of *lasI*
lasI R23W-F	[Phos]TTG*TGG*GCTCAAGTGTTC, with an italicized codon for substitution of Arg for Trp
lasI F28L-F	[Phos]TG*CTG*AAGGAGCGCAAAG, with an italicized codon for substitution of Phe for Leu
lasI F28Y-F	[Phos]TG*TAC*AAGGAGCGCAAAG, with an italicized codon for substitution of Phe for Tyr
lasI W33G-F	[Phos]AGGC*GGC*GACGTTAGTGT, with an italicized codon for substitution of Tyr for Gly
lasI S103A-F	[Phos]TC*GCC*CGTTTCGCCATCA, with an italicized codon for substitution of Ser for Ala
lasI S103E-F	[Phos]TG*GAA*CTCGAGCGTTTC, with an italicized codon for substitution of Ser for Glu
lasI S103V-F	[Phos]TC*GTG*CGTTTCGCCATCA, with an italicized codon for substitution of Ser for Val
lasI-R	ATAAGGCCT**TCA**TGAAACCGCCAGTCGCT, with an underlined *Stu*I restriction site directly downstream of the stop codon (bold) of *lasI*
lasI R23W-R	[Phos]CTTGTGCATCTCGCCCAG, for substitution of Arg for Trp
lasI F28L-R	[Phos]CTTGAGCACGCAACTTGT, for substitution of Phe for Leu
lasI F28Y-R	[Phos]CTTGAGCACGCAACTTGT, for substitution of Phe for Tyr
lasI W33G-R	[Phos]TTGCGCTCCTTGAACACT, for substitution of Tyr for Gly
lasI S103A/V-R	[Phos]GTTCCCAGATGTGCGGCG, for substitution of serine for Ala or Val
lasI S103E-R	[Phos]GATGTGCGGCGAGCAAGG, for substitution of Ser for Glu
Ptac	CGGCTCGTATAATGTGTGGA, primer to sequence multiple cloning site in pME6032
P6032	CCCTCACTGATCCGCTAGTC, primer to sequence multiple cloning site in pME6032
rhlI-F	TATCAATTG**ATG**ATCGAATTGCTCTCTGAAT, with an underlined *Mfe*I restriction site directly upstream of the start codon (bold) of *rhlI*
rhlI F28L-F	[Phos]TC*CTG*ATCGAGAAGCTGG, with an italicized codon for substitution of Phe for Leu
rhlI F28Y-F	[Phos]TC*TAC*ATCGAGAAGCTGG, with an italicized codon for substitution of Phe for Tyr
rhlI W34G-F	[Phos]TG*GGC*GGCGACGTGGTCT, with an italicized codon for substitution of Tyr for Gly
rhlI S103A-F	[Phos]TT*GCC*CGCTACGCCGCCA, with an italicized codon for substitution of Ser for Ala
rhlI S103E-F	[Phos]TGG*GAG*CTTGAGCGCTAC, with an italicized codon for substitution of Ser for Glu
rhlI S103V-F	[Phos]TT*GTG*CGCTACGCCGCCA, with an italicized codon for substitution of Ser for Val
rhlI-R	ATAAGGCCT**TCA**CACCGCCATCGACAGC, with an underlined *Stu*I restriction site directly downstream of the stop codon (bold) of *rhlI*
rhlI F28L-R	[Phos]CCTGATGCCGGTAGCGTC, for substitution of Phe for Leu

rhlI F28Y-R	[Phos]CCTGATGCCGGTAGCGTC, for substitution of Phe for Tyr
rhlI W34G-R	[Phos]GCTTCTCGATGAAGACCT, for substitution of Tyr for Gly
rhlI S103A/VR	[Phos]GCTCCCAGACCGACGGAT, for substitution of Ser for Ala or Val
rhlI S103E-R	[Phos]GACCGACGGATCGCTCGG, for substitution of Ser for Glu

References for Supplementary Table S1.

1. Sambrook, J., & Russell, D.W. Molecular Cloning: A Laboratory Manual (2001) .

2. **Jensen, K. F.** The *Escherichia coli* K-12 "wild types" W3110 and MG1655 have an *rph* frameshift mutation that leads to pyrimidine starvation due to low *pyrE* expression levels. *J Bacteriol* **175:**3401-3407 (1993).

3. **Tavender, T., N. Halliday, K. Hardie, & Winzer, K.** LuxS-independent formation of AI-2 from ribulose-5-phosphate. *BMC Microbiol* **8,** 98 (2008).

4. **Yanisch-Perron, C., J. Vieira, and J. Messing.** Improved M13 phage cloning vectors and host strains: nucleotide sequences of the M13mp18 and pUC19 vectors. *Gene* **33,** 103-119 (1985).

5. **Surette, M. G., M. B. Miller, & Bassler, B. L.** 1999. Quorum sensing in *Escherichia coli*, *Salmonella typhimurium*, and *Vibrio harveyi*: A new family of genes responsible for autoinducer production. *Proc Nat Acad Sci USA* **96,** 1639-1644. (1999).

6. **Heeb, S., C. Blumer, & Haas, D.** Regulatory RNA as mediator in GacA/RsmA-dependent global control of exoproduct formation in *Pseudomonas fluorescens* CHA0. *J Bacterol* **184,** 1046-1056. (2002).

7. **Winson, M., S. Swift, P. Hill, C. Sims, G. Griesmayr, B. Bycroft, P. Williams, & Stewart, G.** Engineering the *luxCDABE* genes from *Photorhabdus luminescens* to provide a bioluminescent reporter for constitutive and promoter probe plasmids and mini-Tn5 constructs. *FEMS Microbiol Lett* **163,** 193-202 (1998).

Supplementary Table S2. Substitutions chosen for mutagenesis of AHL synthases. Residues chosen aligned with those published in (1), and map to equivalent positions in *lasI*. Other substitutions chosen were predicted to insert more structurally conservative changes.

AA position	AA Encoded	WT DNA sequence	Mutant AA	Mutant DNA sequence
lasI				
23	R	CGT	W	TGG
27	F	TTC	L	CTG
			Y	TAC
33	W	TGG	G	GGC
103	S	AGC	A	GCC
			E	GAA
			V	TGC
rhlI				
28	F	TTC	L	CTG
			Y	TAC
34	W	TGG	G	GGC
103	S	TTC	A	GCC
			E	GAG
			V	GTG

References for Supplementary Table S2.

1. Parsek, M. R., A. L. Schaefer, & Greenberg, E. P. Analysis of random and site-directed mutations in *rhlI*, a *Pseudomonas aeruginosa* gene encoding an acylhomoserine lactone synthase. *Mol Microbiol* **26,** 301-310 (1997).

Supplementary Table S3. MTA levels in strains bearing QSSM synthases. Figures for metabolites attained in one representative independent experiment.

	lasI	*rhlI*
Vector alone	(1.03 ± 0.02)	
WT, unmutated gene	26.54 ± 0.43	18.81 ± 0.72
R23W	1.09 ± 0.12	-
F27L; F28L	6.10 ± 0.32	1.89 ± 0.09
F27Y; F28Y	30.38 ± 0.95	1.09 ± 0.12
W33G; W34G	2.04 ± 0.13	1.73 ± 0.03
S103A	43.28 ± 1.06	20.83 ± 0.37
S103E	2.17 ± 0.55	1.98 ± 0.19
S103V	1.29 ± 0.09	21.06 ± 1.84

[†]Concentrations (in relative units) of AMC metabolites were determined by analysing cell content using LC-MS analysis, as described in Materials and Methods. The mean OD_{600} values of triplicate culture samples standard deviations from the means are shown.

Supplementary Table S4. Intracellular concentrations of AMC metabolites decrease in *E. coli* producing active QSSM synthases. Figures for metabolites attained in one representative independent experiment.

Met.	Cell density (OD_{600})	MG1655 [pME6032] –IPTG	MG1655 [pME6032] +IPTG	MG1655 [pME-*lasI*] –IPTG	MG1655 [pME-*lasI*] +IPTG	MG1655 [pME-*rhlI*] –IPTG	MG1655 [pME-*rhlI*] +IPTG
SAM		39.46 ± 0.72	45.67 ± 1.11	43.85 ± 2.25	1.39 ± 0.02	39.77 ± 0.86	23.40 ± 0.29
SAH		0.109 ± 0.001	0.112 ± 0.010	0.102 ± 0.004	0.029 ± 0.004	0.086 ± 0.002	0.058 ± 0.006
SRH		0.590 ± 0.025	0.561 ± 0.016	0.564 ± 0.007	0.060 ± 0.004	0.599 ± 0.051	0.444 ± 0.064
HCY		8.204 ± 0.819	8.250 ± 0.486	8.614 ± 0.291	2.109 ± 0.268	8.834 ± 1.360	6.360 ± 0.216
MET		188.539 ± 5.668	182.668 ± 11.185	180.537 ± 3.735	127.390 ± 4.525	185.038 ± 12.581	146.284 ± 4.002

†Concentrations (in relative units) of AMC metabolites were determined by analysing cell content using LC-MS analysis, as described in Materials and Methods. The mean OD_{600} values of triplicate culture samples standard deviations from the means are shown.
